# Role of Growth Factors in the Pathogenesis of Systemic-Sclerosis-Associated Fibrosis

**DOI:** 10.3390/ijms26199596

**Published:** 2025-10-01

**Authors:** Fabian A. Mendoza, Sonsoles Piera-Velazquez, Sergio A. Jimenez

**Affiliations:** 1 Jefferson Institute of Molecular Medicine, and Scleroderma Center; Thomas Jefferson University, Philadelphia, PA 19107, USA; 2 Division of Rheumatology, Department of Medicine, Thomas Jefferson University, Philadelphia, PA 19107, USA

**Keywords:** systemic sclerosis, pathogenesis, fibrosis TGF-β, PDGF, FGF, VEGF, caveolin, lysophosphatidic acid, Wnt

## Abstract

Systemic Sclerosis (SSc) is a systemic autoimmune disease of unknown etiology characterized by a severe fibroproliferative vasculopathy and frequently progressive cutaneous and internal organ fibrosis. The small-vessel vasculopathy and the tissue fibrotic alterations are responsible for the most serious clinical and pathological manifestations of the disease and for its high mortality. Despite the high severity and frequent mortality, there are currently no optimal therapeutic approaches for SSc, and its complex pathogenesis has not been fully elucidated. Numerous studies have suggested that growth factors and related regulatory macromolecules released from inflammatory and other cells present in the affected tissues play a crucial role in the frequently progressive cutaneous and visceral fibrosis. Here, we will review some of the recent studies describing the role of various growth factors and related macromolecules in the development and progression of the fibrotic process in SSc.

## 1. Introduction

Systemic Sclerosis (SSc) is a clinically heterogeneous systemic autoimmune disease of unknown etiology characterized by a frequently progressive fibrotic process affecting the skin and various internal organs. The fibrotic process in SSc is usually accompanied by endothelial cell alterations and severe occlusive fibroproliferative vasculopathy of small arteries and arterioles, the presence of a chronic inflammatory process in the affected tissues, and the occurrence of humoral and cellular immune abnormalities [1,2,3,4]. Numerous studies have provided substantial information about the intricate and complex mechanisms and the altered regulatory pathways involved in SSc pathogenesis. However, the entire process has not been completely elucidated [5,6,7,8,9].

**Figure 1:**
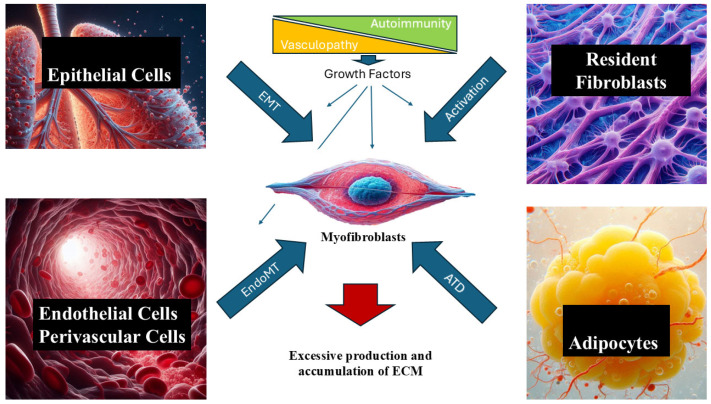
**Pathophysiology of SSc.** Early events, including endothelial cell dysfunction, vasculopathy, and alterations in the immune process in the affected tissues, cause increased production and secretion of multiple growth factors with autocrine, paracrine, and long-distance effects. These growth factors promote the activation and transformation of resident fibroblasts and transdifferentiation of multiple cell lines, including epithelial cells (EMT), endothelial cells (EndoMT), and adipocytes (AMT) into myofibroblasts. Myofibroblasts are critical regulators of the fibrotic process. They are able to synthesize high amounts of various interstitial collagens and other fibrotic extracellular matrix (ECM) macromolecules that deposit in the skin and various internal organs, causing target organ dysfunction.

The most important and characteristic clinical and pathologic manifestations of the disease result from an often-progressive fibrotic process that causes the exaggerated and abnormal deposition of various interstitial collagens and other fibrotic extracellular matrix (ECM) macromolecules in the skin and various internal organs. The molecular mechanisms involved in the initiation and progression of SSc-associated tissue fibrosis have not been fully elucidated. However, it has become apparent that cellular transdifferentiation events causing the phenotypic conversion of various cell types, including resident fibroblasts and epithelial, endothelial, perivascular, and adipose tissue cells into activated myofibroblasts, the cellular elements ultimately involved in the exaggerated and excessive production and accumulation of fibrotic tissue, play a crucial role [10,11,12,13,14,15,16]. Myofibroblasts, crucial fibrosis mediators in SSc, are mesenchymal cells that display a markedly fibrogenic phenotype characterized by a persistent and exaggerated expression of genes encoding various interstitial collagens and other ECM proteins, the downregulation of genes for matrix-degrading enzymes, and the initiation of expression of contractile proteins such as α-SMA [17,18,19]. Their elevated biosynthetic functions are maintained for serial passages in vitro, indicating that these cells display fundamental molecular alterations leading to a persistent dysregulation of expression of multiple genes that encode the macromolecules involved in the development of SSc-associated tissue fibrosis [17,18,19,20,21,22,23,24]. A summary of the pathophysiology of SSc is displayed in Figure 1.

Here, we will review recent developments in the role of growth factors and of several related regulatory pathways, including the morphogen proteins Wnt, Hedgehog, and Notch, in the pathogenesis of the SSc-associated fibrotic process, focusing on the transdifferentiation of various cell types into activated myofibroblasts, and we will briefly describe the molecular mechanisms involved in these effects. These growth factors and signaling pathways are particularly crucial because they act as central upstream regulators of myofibroblast activation and ECM production, orchestrating the persistent fibrotic responses characteristic of SSc. Understanding their roles provides critical insight into potential therapeutic targets for modulating fibrosis in this disease. The numerous and extensive publications dedicated to these topics preclude us from including in this review all the related studies, and we express our deepest apologies to the investigators whose work was not specifically discussed.

## 2. Role of Growth Factors in SSc-Associated Tissue Fibrosis

Multiple recent studies have examined and described the cellular and molecular mechanisms involved in the fibrotic process (reviewed in refs. [25,26,27,28]). Numerous studies have shown the crucial role of polypeptide growth factors in the development and extension of the SSc-associated fibrotic process (reviewed in ref. [29]). The most commonly implicated growth factors include transforming growth factor beta (TGF-β), connective tissue growth factor (CTGF), platelet-derived growth factor (PDGF), fibroblast growth factors (FGF), vascular endothelial growth factor (VEGF), and insulin-like growth factor (FGF).

### 2.1. Transforming Growth Factor Beta (TGF-β)

It is generally considered that TGF-β is the most important growth factor involved in the initiation and progression of the fibrotic process [29,30] and that it plays a crucial role in SSc-associated tissue fibrosis [29,31,32,33,34]. There are three distinct TGF-β isoforms, TGF-β1, TGF-β2, and TGF-β3, that are encoded by three different genes with different promoters and regulators. Our current understanding of this differential regulation is limited. Although all isoforms binds to the same receptor, they display differences in their levels of expression in various tissues, as well as in their specific molecular and cellular effects [30]. In general, TGF-β1 and TGF-β2 are considered strongly profibrotic and follow the mechanistic explanation described in this section, but TGF-β3 has been postulated to be at least less pro-fibrotic than the other isoforms. Conversely, selective TGF-β3 blockade has been postulated to attenuate fibrosis with a favorable safety profile compared with pan-TGF-β inhibition [35,36], but differences in the effects of TGF-β3 on fibrosis in in vivo and in vitro studies point out that its effect is highly context-dependent, and further research is needed.

There are three types of TGF-β receptors, TβRI, TβRII, and TβRIII, expressed differentially in different cells. TβRI and TβRII display a high affinity for TGFβ1 and low affinity for TGFβ2. TβRIII has a high affinity for both homodimeric TGFβ1 and TGFβ2 [37]. The TβR are transmembrane receptors, with serine/threonine kinase properties, that, after activation, will cause phosphorylation of various intracellular mediators.

### 2.2. Receptor Activation and Intracellular TGF-β Pathways

Classic activation of its receptors by TGF-β has been extensively reviewed. Briefly, following synthesis, TGF-β1 is secreted along with the latency-associated peptide (LAP). Both are maintained together by non-covalent bonds. The LAP prevents TGF-β from its direct interaction with its receptors, and it binds to the extracellular matrix (ECM) [38,39]. After cleavage by proteases (such as MMPs), or after an alternative interaction of an integrin receptor (such as αvβ6 receptor in fibroblasts) [39], which cause separation by mechanical forces [40], TGF-β binds to TβRII, which is expressed in all human cells. This ligand binding recruits the co-receptor TβRI, a specialized receptor called activin receptor-like kinase (ALK). The extracellular binding domain of ALK displays a glycine- and serine-rich sequence (GS) domain. This GS domain is activated by phosphorylation by the TGF-βRII. Following phosphorylation, the TGF-βRI forms heterodimers with TβRII and becomes functionally activated [41,42,43]. TGF-β receptors may have different ALK domains, expressed in different cells. For example, the activin receptor-like kinase 5 (ALK5) is widely expressed in fibroblasts and is responsible for the pro-fibrotic intracellular processes described in detail below, but TGFβ1 in endothelial cells signals through ALK1, causing phosphorylation of Smad 1, 5, and 8, affecting neoangiogenesis [41,44]. It is believed that this structural variation can be associated with TGF-β organ-specific functions. Isotypes of TβRII and different ALK-TβRI receptors have been described in different human cell types, and early studies support an important role in health and disease [41].

**Figure 2:**
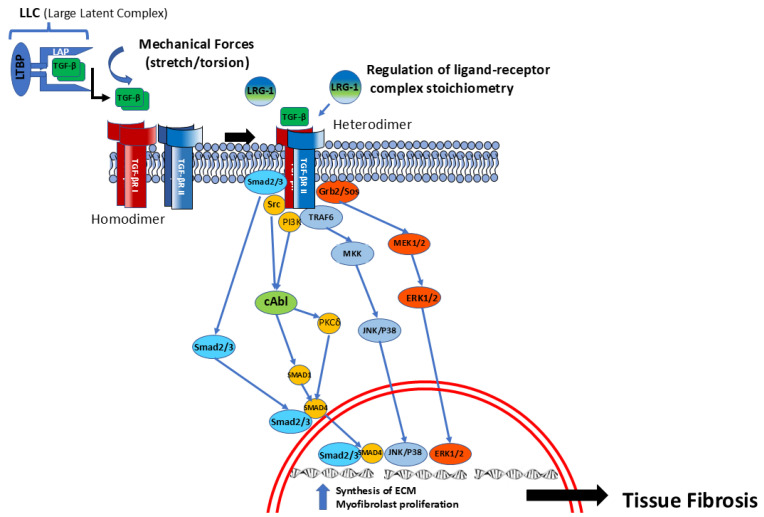
**TGF-β intracellular pathways.** Following release of TGF-β from its large latent complex, TGF-β links with its receptor, causing heterodimerization and activation of two main different pathways: (1) The canonical pathway is that in which the heterodimeric TGF-β receptor phosphorylates Smad2 and Smad3, which will form a complex with Smad4, allowing for nuclear translocation and inducing transcription of ECM molecules and other myofibroblast-activating molecules. (2) The non-canonical pathway is that in which the activated TGF-β receptor induces phosphorylation of tyrosine residues that are able of recruiting Grb2/Sos to activate Erk through Ras, Raf, and MAPK cascades. Intranuclearly, Erk regulates target gene transcription in conjunction with Smads, resulting in increased ECM production. Another very relevant non-canonical pathway is activated by the TGF-β receptor phosphorylation of Src and stimulates a fibrotic response mediated by C-Abl/Smad pathways.

Once activated, TβRI (ALK) phosphorylates Smad2 and Smad3, which, following phosphorylation, bind with the co-Smad, Smad4, allowing for the translocation of this complex into the nucleus, modulating the expression of pro-fibrotic target genes [42,45,46,47,48,49,50,51,52]. These pathways are represented in Figure 2 and are known as the TGF-β canonical pathway. In parallel to these canonical pathways, TβRI also activates tyrosine kinases, named the non-canonical pathway, including JAK/STAT, MAPK ERK, and c-ABL [51] (Figure 2).

### 2.3. TGF-β Pleiotropic Profibrotic Effects

The potent pro-fibrotic effects of TGF-β are mediated by multiple mechanisms that include the potent stimulation of expression of genes encoding various interstitial collagens and other ECM macromolecules by fibroblasts and other mesenchymal cells, the reduction in collagen-degrading metalloproteinases, and the increase in the production of broad-spectrum protease inhibitors such as tissue inhibitor of metalloproteinase 1 [29,30,36,37]. Other crucial effects of TGF-β that are of great relevance to the fibrotic process involve the induction of cellular transdifferentiation of various types of cells, including resting fibroblasts, epithelial and endothelial cells, and adipocytes into activated myofibroblasts, displaying a marked increase in the expression of multiple pro-fibrotic genes, including α-SMA, and various interstitial collagens (reviewed in ref. [16]).

TGF-β also sensitizes fibroblasts to its own effects, maintaining them in a persistently activated state, thus creating a vicious cycle involving an autocrine mechanism that causes further production of TGF-β. Furthermore, these effects display a remarkable context-dependent variability [38] and have been described with various TGF-β isoforms (TGF-β1,2) [53]. Recent studies regarding the overall pro-fibrotic TGF-β effects indicated that the tissue matrix stiffness was capable of modulating TGF-β protein activation [39,40]. Subsequent characterization of the modulation of TGF-β activity induced by substrate and tissue mechanical properties demonstrated that there was decreased expression of collagen I, collagen III, and matrix metalloproteinase 2 (MMP2) when cells were cultured on compliant substrates compared to stiffer ones [40].

### 2.4. Novel TGF-β-Regulatory Pathways

Although most studies have focused on the TGF-β signaling events in the control of ECM production and remodeling, it has become apparent that numerous additional molecular pathways may participate in the regulation of the pro-fibrotic effects of TGF-β. For example, recent studies have shown that several of the fibrogenic transcriptional responses to TGF-β involve early growth response transcription factors [54] and require activation of the focal adhesion kinase/Src (FAK/Src) molecular cascade. It was further shown that the TGFβ-activated kinase 1 (TAK1) acts downstream of FAK/Src to mediate the TGF-β pro-fibrotic responses. Consequently, TGF-β-induced JNK phosphorylation is impaired in the absence of TAK1, inhibiting the expression of mRNA for α-SMA and other TGF-β-induced pro-fibrotic genes [55].

Important TGF-β functions, including some fibrosis-regulatory effects, have been shown to be mediated by non-Smad pathways that contribute to the fibrosis and vasculopathy of skin and internal organs in SSc [51,56,57]. Some of these involve various non-receptor tyrosine kinases (reviewed in ref. [57]), including the cytoplasmic Abelson kinase (c-Abl) and protein kinase C-δ (PKC-δ). One crucial pro-fibrotic pathway involves the participation of c-Abl in TGF-β-induced transdifferentiation of endothelial cells into activated myofibroblasts, an effect mediated by the cooperative interaction with PKC-δ [58].

There are several other recently identified regulatory pathways that may be involved in the overall TGF-β effects. Extensive studies have shown that periostin, a widely distributed matricellular protein, plays a key role in the pathogenesis of the SSc fibrotic process [59]. This study examined serum periostin levels and skin tissue expression in patients with diffuse and limited SSc in comparison with healthy controls. The results showed significantly elevated periostin expression in SSc skin, and these levels were even higher in affected skin samples from patients with early (less than 5 years) diffuse SSc. A significant correlation was found with the severity of the skin fibrosis score. A more recent study [60] determined the periostin levels in serum from 35 patients with established SSc, including diffuse and limited clinical SSc subsets, 15 patients with very early SSc diagnosis (VEDOSS), and 30 sex-matched healthy controls. The results showed higher periostin serum levels in all SSc patients compared to normal controls, and there were no significant differences between the diffuse, limited, and very early SSc groups. However, the periostin levels were substantially higher in patients with digital ulcers. In this study, skin periostin expression was examined by immunohistochemistry and found in all samples from affected SSc skin and in a large number of samples from non-affected skin from SSc patients, results that were in contrast with a remarkable absence of periostin immunostaining in normal skin samples [60].

Periostin effects have been shown to be mediated by a marked amplification of the TGF-β intracellular signals [61]. A more recent study [62] demonstrated high expression of SOX11 in cultured SSc dermal fibroblasts and that these levels were markedly increased by treatment of the cells with TGF-β. It was further shown that SOX11 and periostin established a highly specific molecular complex that was responsible for the activation of TGF-β signals in the development of cutaneous fibrosis [62].

Another pathway regulating TGF-β signaling involves some members of the Leucine-rich α2-glycoprotein (LRG) family of leucine repeat proteins. These proteins are expressed in multiple cells and tissues and are involved in complex regulatory pathways, including signal transduction, cell adhesion, and development. Leucine-rich alpha-2-glycoprotein 1 (LRG1) has been shown to be one of the most important LRG components in the fibrotic process, causing an increase in expression levels of COL1A1 and COL1A2 mRNA and a decrease in matrix metalloproteinase 1 (MMP-1) mRNA levels. These effects appeared to be related to LRG1 modulation of the overall effects of TGF-β in fibroblasts [63]. Although the mechanisms of the interactions of LRG1 with TGF-β have not been fully elucidated, recent studies have suggested that LRG1 regulates the stoichiometry of the TGF-β receptor complex, causing activation of Smad/TGF-β canonical pathways. It was further shown that these effects were mediated through the induction of Smad2 phosphorylation and resulted in a potent increase in collagen production and ECM deposition [63].

Under normal physiological conditions, LRG1 plays a role in the regulation of immune responses and tissue neovascularization, however, under pathological conditions, it may be an important component of the development of various clinical disorders including tissue fibrotic responses. Indeed, it has been shown that LRG1 plays a role in the development of pulmonary and renal fibrosis [64,65]. Although the precise role of LRG1 in the fibrogenic and vascular alterations characteristic of SSc has not been studied in detail, it has been shown that LRG1 pro-fibrotic effects include the in vitro stimulation of expression of genes encoding pro-fibrotic molecules in cultured fibroblasts and the marked reduction in the extent of bleomycin-induced tissue fibrosis in LRG1 KO mice [64], and it has been recently suggested that LRG1 levels may serve as a biomarker of the extent and severity of the SSc fibrotic process and, specifically, for SSc-associated ILD [66].

### 2.5. Connective Tissue Growth Factor (CTGF)

CTGF, also known as cellular communication network-2 (CCN2), is another pleotropic growth factor described initially to be secreted by vascular endothelial cells [67], and it has emerged as an important mediator of normal and pathological tissue fibrotic responses. TGF-β causes potent stimulation of CTGF synthesis in fibroblasts, vascular smooth muscle cells, and endothelial cells. Numerous studies have suggested that CTGF may represent a downstream mediator of TGF-β fibrogenic effects [68], and it has been considered to play a crucial role in the SSc fibrotic process [69,70]. One study showed that serum CTGF levels were increased in SSc patients with more severe disease and that these levels correlated with the extent of skin sclerosis and the severity of pulmonary fibrosis [70]. An assessment of CTGF expression in SSc tissues demonstrated strong CTGF signals in affected SSc skin fibroblasts, whereas there was no expression in the skin from normal controls [71]. CTGF has also been found to be overexpressed in lung fibroblasts isolated from SSc patients. Furthermore, it was demonstrated that CTGF induced remarkable changes in the lung fibroblast proteome. These studies identified novel CTGF-responsive molecules that may play important roles in lung tissue repair and pathologic fibrosis. One of these molecules, IQ-motif-containing GTPase-activating protein (IQGAP1), was significantly elevated in lung fibroblasts from patients with SSc-associated pulmonary fibrosis, and it was suggested that it may serve as a molecular marker for this group of patients [72].

**Figure 3:**
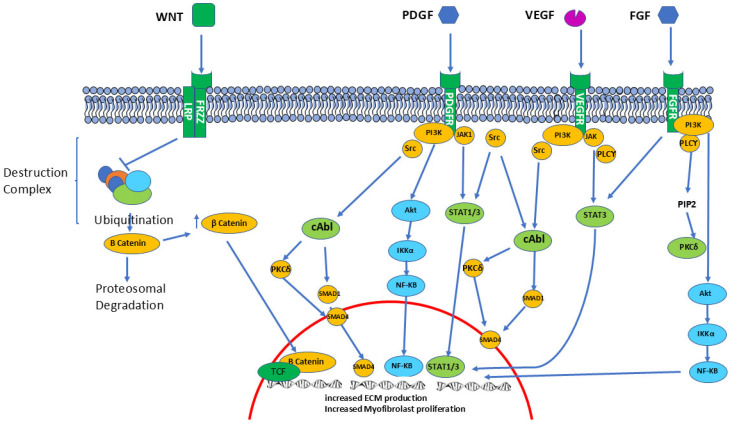
**Intracellular pathways of selected pro-fibrotic molecules.** Schematic intracellular pathways of various intracellular mediators of fibrosis, including Wnt, PDGF, VEGF, and FGF. Wnt promotes inhibition of proteosomal degradation of β Catenin, whereas PDGF, VEGF, and FGF are tyrosine kinases receptors that phosphorylate multiple intracellular mediators to promote a pro-fibrotic cellular state.

Analysis of the extensive CTGF signaling pathways (see Figure 3) indicated that it interacts with a large number of molecules, including cell surface receptors, epidermal growth factor, and various ECM proteins, resulting in a very broad range of regulatory effects and a multiplicity of cellular functions [73,74]. Some of these effects are of substantial relevance to SSc pathogenesis owing to the fact that vascular wall smooth muscle cells are among the main targets for CTGF modulation [75]. These observations have suggested that CTGF may play a crucial role in the development of Raynaud’s Phenomenon and other vascular alterations that are characteristic of SSc. Mechanistic studies have shown that the N-terminal domain of the molecule mediates the cellular transdifferentiation effects, whereas the C-terminal domain regulates fibroblast proliferation [76]. However, given the fact that the function of CTGF is context-dependent and may vary depending on the type of tissue and cells, we still only have a partial and fragmented understanding of CTGF interactions and function [77].

### 2.6. Platelet-Derived Growth Factor (PDGF)

Numerous studies have shown that members of the PDGF family of growth factors play an important role in the maintenance of normal connective tissue homeostasis and that structural or functional alterations to these molecules may be involved in the pathogenesis of multiple diseases, including SSc (reviewed in ref. [78]). PDGF is secreted by fibroblasts, endothelial cells, platelets, macrophages, and other types of cells. Its effects are initiated by the activation of two distinct receptor tyrosine kinases (PDGFR-α and PDGFR-β) that induce a potent mitogenic stimulation of vascular smooth muscle cells and dermal fibroblasts. Elevated expression of PDGF and its receptors have been found in SSc skin and lung tissues, and there is evidence that TGF-β stimulates the expression of the PDGFR-α receptor in SSc cells, suggesting that cross-talk between TGF-β and PDGF pathways may regulate tissue fibrosis in SSc [78]. Several studies support the role of PDGF and TGF-β1 in the pathogenesis of SSc lung disease, including the observations of elevated levels of these growth factors in bronchoalveolar lavage fluid from affected SSc patients [79]. Furthermore, the potent smooth-muscle-cell-mitogenic effects of PDGF have been implicated to play a role in the severe pulmonary vasculature fibroproliferative alterations occurring during the development of primary and SSc-associated pulmonary arterial hypertension [80].

A highly novel pathway for the pro-fibrotic effects of PDGF has recently been identified following the demonstration that PDGFR-α is a target of specific autoantibodies produced by some SSc patients that are detectable in purified immunoglobulins isolated from the serum of these patients [81]. It has further been shown that binding of these antibodies to their corresponding receptors on the fibroblast cell surface induces activation of quiescent fibroblasts, resulting in the induction of potent pro-fibrotic effects in these cells. Selective activation of the Ha-Ras-ERK1/2 signaling pathway and the stimulation of reactive oxygen species (ROS) has been shown to drive these pro-fibrotic effects [82,83]. It has also been suggested that activation of intracellular molecular cascades by specific autoantibodies against PDGFR-α might stimulate and sustain the cellular transdifferentiation process, resulting in their conversion to a myofibroblast phenotype. Collectively, these effects result in the persistent pro-fibrotic activation of SSc fibroblasts and indicate that the PDGF/PDGFR pathways may represent important and relevant targets for anti-fibrotic therapy in SSc [82,83].

### 2.7. Fibroblast Growth Factors (FGFs)

FGFs comprise a large family of polypeptide growth factors characterized by their ability to induce potent mitogenic effects in numerous cell types. FGFs play multiple important roles during development, angiogenesis, and wound healing [84,85]. Numerous studies have demonstrated the fibroblast-mitogenic effects of FGFs during inflammatory and fibrotic responses, often potentiating the pro-fibrotic effects of TGF-β, although some recent studies have described controversial results indicating that some of the members of the FGF family may cause antifibrotic effects that may be mediated by inhibition of TGF-β pathways [86,87]. Regarding the role of FGFs in SSc pathogenesis, it has been demonstrated that basic FGF (FGF-2) is increased in the skin of SSc patients [88]. However, the precise role of FGFs in the initiation of progression of the fibrotic process in SSc has not been completely elucidated, and further studies will be required to conclusively determine the contribution of these potent growth factors to the pathogenesis of fibrosis in SSc.

### 2.8. Vascular Endothelial Growth Factor (VEGF)

VEGF is an endothelial-cell-specific growth factor with multiple functions that include stimulation of endothelial cell proliferation and differentiation and modulation of endothelial permeability [89,90,91]. The expression of VEGF is highly regulated, and it is markedly induced by hypoxia. Based on the demonstration that molecular effects caused by tissue hypoxia are prominent pathologic alterations in affected skin and other organs of patients with SSc, it has been postulated that VEGF dysfunction may play an important role in the development of the angiogenesis abnormalities characteristic of the disease [92,93,94]. Measurement of serum levels of VEGF in patients with SSc and healthy controls showed that serum VEGF levels were significantly higher in SSc patients and correlated with the extent of skin sclerosis and nailfold capillary loss, supporting the notion that high VEGF levels may participate in the capillary damage in SSc and may correlate with the extent and severity of the fibrotic process and the disturbed angiogenesis characteristic of SSc [92,93,94]. Furthermore, given the potent proangiogenic effects of VEGF, it has been suggested that it may play an important role in the pathogenesis of SSc-associated vascular fibroproliferative alterations such as PAH [95]. In support of this notion, an early study described increased serum VEGF levels in SSc patients with PAH [96]; however, some subsequent studies failed to confirm this correlation [97].

The apparently conflicting results of VEGF serum or tissue levels from SSc patients can be explained by the fact that VEGF 165b, an alternative splicing variant of VEGF-A, is overexpressed in SSc endothelial cells, fibroblasts, and also in peripheral blood mononuclear cells. The VEGF 165b isoform, as opposed to the most common VEGF 165 isoform, displays potent antiangiogenic effects due to defective phosphorylation of intracellular tyrosin kinases [98]. Consequently, quantification of VEGF without considering the presence of isoforms and its activity may lead to different conclusions.

### 2.9. Insulin-like Growth Factors (IGFs)

IGFs, initially described as a serum factor that stimulated sulfate incorporation by cartilaginous tissues, comprise a family of regulatory polypeptides with high sequence similarity to insulin that are involved in numerous physiologic states, including growth and development, cellular proliferation and apoptosis, and aging [99,100]. Several studies have examined the role of IGFs and IGF-binding proteins (IGFBPs) in fibrotic conditions, including pulmonary fibrosis and SSc. Serum IGF1 and IGFBP3 levels in patients with SSc were significantly elevated and correlated with the extent of skin involvement and the presence of pulmonary fibrosis [101]. Furthermore, IGF1 mRNA was upregulated in the affected skin of patients with SSc. The extensive studies of Feghali-Bostwick and collaborators examined the pro-fibrotic role of IGF2 in normal fibroblasts and in normal and fibrotic skin and lung tissues [102,103]. These studies demonstrated that IGF2 is a potent inducer of collagen production and other fibrotic pathways and that these effects are mediated by several distinct mechanisms, including increasing the expression of pro-fibrotic signaling molecules TGFβ-2 and TGFβ-3, increasing the activity of various enzymes involved in collagen post-translational modifications such as the prolyl and lysyl hydroxylases, and decreasing the expression of several enzymes involved in collagen degradation. Other studies from the same investigators demonstrated that the levels of SOX9, a transcription factor that has recently been shown to be involved in the development of the SSc fibrotic process, are increased by IGF2 in lung tissues and fibroblasts [98]. These studies also revealed increased IGF2 expression in fibroblastic foci of SSc lungs and cultured primary SSc lung fibroblasts and showed that IGF2 induced a dose- and time-dependent increase in collagen type I and fibronectin production and triggered the activation of several important kinase pathways, including the PI3K signaling cascade in these cells. These results provided strong support and novel insights into the role of IGF2 in the pathogenesis of fibrotic disorders [104].

## 3. Other Regulatory Pathways Involved in the SSc Fibrotic Process

Besides the pathways mediated by TGF-β and other growth factors discussed above, alterations in several other regulatory mechanisms are very likely important components of the complex sequence of events in the pathogenesis of SSc-associated tissue fibrosis. These will be discussed briefly in the following sections.

### 3.1. PKC-Delta

Another important kinase involved in the non-Smad TGF-β signaling pathway is PKC-δ, a serine- and threonine-specific protein kinase that plays a critical role in numerous immunological responses, cytokine signaling, and host defense mechanisms. The role of PKC-δ in the pathogenesis of fibrotic diseases, including SSc, has been extensively investigated. Studies from our laboratories showed that dermal fibroblasts from SSc patients contain higher PKC-δ levels than normal cells, and further studies demonstrated that PKC-δ displayed strong effects on the transcriptome of normal and SSc fibroblasts, causing a potent inhibition of type I collagen production and abrogation of TGF-β-induced stimulation of collagen gene expression in normal human dermal fibroblasts [105,106]. Some of these PKC-δ effects are specifically relevant to the regulation of TGF-β1-induced target gene expression involving the Smad proteins in a variety of cell types, as it has been shown that TGF-β requires PKC-δ to activate several of its target genes in a variety of cells, including dermal and pulmonary fibroblasts, vascular smooth muscle and endothelial cells, and even mesangial cells [107].

Other studies examined the role of PKC-δ on the expression of the gene encoding the α2 collagen chain (COL1A2) and demonstrated that PKC-δ regulation of the gene occurs at the level of the gene promoter and involves complex interactions between Sp1 and Fli1, the Friend leukemia integration-1 transcription factor. These studies indicated that TGF-β induces phosphorylation of PKC-δ and that phosphorylated PKC-δ, in turn, phosphorylates Fli1 at threonine 312, and it was shown that this step is essential for the TGF-induced increase in the collagen gene transcriptional activity [108,109].

### 3.2. P13-Kinase

Other important signaling pathways are initiated by TGF-β activation of phosphoinositide 3-kinases (PI3Ks). The PI3Ks phosphorylate inositol-containing lipids to yield phosphoinositol 3-phosphate. The phosphorylated inositol-3-phosphate plays critical roles in the regulation of multiple cellular functions following binding to specific lysophosphatidic acid (LPA) receptors that are expressed in various types of cells, including cells of the immunologic system, and alterations in the regulatory pathways mediated or involving PI3Ks have been described in several human diseases [106]. The potential role of PI3K in the pathogenesis of tissue fibrosis in SSc has been supported by studies that showed elevated PI3K activity in platelet lysates from SSc patients [107] and demonstrated a potent effect in the regulation of expression of the α2(I) collagen gene in normal and SSc fibroblasts [108]. Subsequent studies have described the important role of the rac/PI3K pathway in the differentiation and activation of myofibroblasts through endothelin receptor (ETA) in lung SSc fibroblasts [109].

### 3.3. Lysophosphatidic Acid

Lysophosphatidic acid (LPA) is a phospholipid derivative that has undergone hydrolysis to remove one acyl group. LPA, along with other related molecules, can act as a signaling molecule. LPA is a potent mitogen activator of high-affinity G-protein-coupled receptors called LPARs (formerly known as EDGs) [110,111]. Following LPARr activation, the small GTPase Rho is activated, subsequently activating Rho kinase. This can lead to the formation of stress fibers and cell migration by inhibiting myosin light-chain phosphatase. Furthermore, RhoA/ROCK pathway links cytoskeletal tension (ECM stiffness) with TGF-β profibrotic signaling, promoting myofibroblast differentiation and activation [112].

In serum from SSc patients, LPA has shown higher concentration levels compared to normal controls [113], and a recent study has described a pro-fibrotic amplification loop involving LPA and interleukin-6 (IL-6) that activated SSc fibroblasts [114,115]. These studies indicated that LPA plays an important role in SSc-associated tissue fibrosis and has raised strong interest in the study of LPA inhibition as a potential SSc therapeutic intervention. Indeed, several studies have been performed to test this hypothesis. The study by Ledein et al. [114] examined the effects of the selective LPA inhibitor SAR100842 on LPA-induced activation of SSc dermal fibroblasts and skin biopsies. The results demonstrated potent inhibition of LPA1-induced inflammatory and pro-fibrotic effects. In a related clinical trial, Allanore et al. studied SAR100842 administered orally and performed a double-blind placebo-controlled study in 15 dcSSc patients lasting 8 weeks [116]. The inhibitor was well tolerated and caused an improvement in skin involvement compared with the placebo, although the difference did not reach statistical significance. However, a gene signature analysis suggested that the expected therapeutic effects had been accomplished. These results are highly promising but might also be explained by the large proportion of subjects receiving background immunosuppressive medications. Therefore, subsequent validation in a larger controlled trial will be required.

A similar study was a phase IIa placebo-controlled trial in patients with dcSSc treated with Ziritaxestat, a selective LPA synthesis inhibitor. The results showed that the inhibitor was significantly more effective than the placebo in improving skin involvement following 24 weeks of treatment [117]. Blood biomarker analysis supported the clinical assessment, showing that Ziritaxestat lowered the levels of fibrosis-associated biomarkers. Remarkably, the results suggested that Ziritaxestat provided additional beneficial effects, including a reduction in expression of several genes associated with inflammation, oxidative phosphorylation, and abnormalities in mitochondrial function. Although the results were highly encouraging and Ziritaxestat administration was well tolerated, these results also required confirmation in a larger and well-powered clinical trial. Unfortunately, two mirror phase III studies evaluating the efficacy and safety of Ziritaxestat in IPF (NCT03711162 and NCT03733444) were halted after reaching criteria of futility in the rate of decline of forced vital capacity (FVC). This cancellation also affected the clinical development for SSc patients [118].

HZN-825 (Fipaxalparant) was investigated in a phase II clinical trial (NCT04781543), demonstrating tolerability and safety, but no significant change in its primary (FVC) or secondary outcomes caused termination after meeting pre-defined criteria for futility [119].

Despite the initial enthusiasm for the use of LPA inhibitors being halted by disappointing IPF data, inhibition of LPA (and its downstream Rho-ROCK pathway) is still an attractive potential antifibrotic target for SSc.

### 3.4. Caveolin-1-Mediated Regulation

Another recently identified mechanism of regulation and fine tuning of TGF-β activity involves caveolin-1, the most important member of a family of proteins found in lipid rafts that play key regulatory roles in multiple cellular functions, including TGF-β signaling and tissue fibrotic responses [120,121,122]. Extensive studies have shown that TβRs are internalized both by caveolin-1-associated lipid rafts and by early endosome antigen 1 (EEA-1) non-lipid-raft pathways. Non-lipid-raft-associated internalization increases TGF-β signaling, whereas caveolin-associated internalization increases TβR degradation, thereby effectively decreasing or abolishing TGF-β signaling. Further studies have shown that the TβRs contained in the EEA-1 positive compartment are responsible for downstream Smad activation. In contrast, TβRs present in caveolin-1 containing lipid rafts caused recruitment of Smurf/Smad7 with subsequent receptor ubiquitination and rapid degradation and turnover [123,124,125]. This is a novel mechanism of regulation of TβR function. Therefore, a reduction in caveolin-1 would result in uncontrolled activation of all TGF-β-mediated pathways, including those responsible for SSc-associated tissue fibrosis. These studies provided strong support for the concept that caveolin-1 plays an important role in the fibrotic process associated with SSc [125,126,127]. The concept that caveolin-1 plays an important role in SSc-associated tissue fibrosis is supported by the observation that caveolin-1 reduction in vitro induced the development of endothelial-to-mesenchymal transition in murine lung endothelial cells [128]. Related studies showed that restoration of caveolin-1 function employing cell-permeable peptides prevented the development of experimentally induced pulmonary hypertension and right-ventricular hypertrophy in rats, and several other studies described results suggesting that restoration of caveolin function may represent a novel approach for treatment of fibrotic diseases, including SSc and pulmonary fibrosis [129,130].

### 3.5. Janus Kinases (JAK) and Signal Transducer and Activator of Transcription (STAT)

The JAK proteins are a family of highly active non-receptor tyrosine kinases that, along with the STAT proteins, are key mediators of intracellular signaling for multiple cytokines and interferons and that are involved in the development of fibrotic diseases, including SSc [131]. The JAK/STAT pathway plays a double role in SSc pathogenesis: (1) several of its molecular components are crucial downstream effectors of cytokines strongly associated with fibrotic responses such as the interleukins IL-4, IL-6, and IL-13; and (2) the pathway may act as a TGF-β downstream effector contributing to the phenotypic differentiation of fibroblasts into myofibroblasts and may also amplify TGF-β signaling by increasing the expression and cellular levels of TGF-β.

The extensive study by Dees et al. [132] evaluated the role of JAK2 in the pathogenesis of SSc and examined the effects of JAK2 inhibition on the TGFβ-dependent stimulation of the expression of pro-fibrotic genes in cultured SSc dermal fibroblasts. This study demonstrated increased activation of JAK2 and STAT3 in the skin of SSc patients, which persisted in cultured SSc fibroblasts. The selective JAK2 inhibitor, TG 101209, reduced basal collagen synthesis in SSc fibroblasts and abrogated the stimulatory effects of TGF-β. The antifibrotic effects of JAK2 inhibition were validated in vivo in a bleomycin and in a TSK animal model of experimental fibrosis. Collectively, the results of these studies had direct translational implications supporting the use of JAK2 inhibitors as potential agents for the treatment of tissue fibrosis in SSc and other fibrotic diseases [133,134].

### 3.6. Peroxisome-Proliferator-Activated Receptors (PPAR)

The PPAR proteins comprise a large number of nuclear receptors that play various roles in the regulation of lipid metabolism but also modulate several important immunologic and inflammatory pathways [135,136]. These receptors have been classified into three distinct species (α, β, γ) according to their functions and molecular structures. PPAR-γ is a broadly expressed nuclear orphan receptor originally identified in adipose tissue that plays a crucial role in glucose and lipid metabolism. Recent studies have described novel PPAR-γ functions affecting the regulation of TGF-β effects on connective tissue homeostasis and tissue fibrotic responses, including SSc-associated fibrotic alterations [137,138,139,140]. Furthermore, it was shown that a decrease in PPAR-γ expression in mice resulted in an exaggerated fibrotic response to bleomycin and induced an accumulation of activated myofibroblasts [139]. Given these important antifibrotic effects of PPAR-γ, several studies have examined the possible antifibrotic effects of PPAR-γ agonists and have suggested that drugs targeting this pathway may be effective for the control of SSc-associated fibrosis [141,142].

## 4. Regulation by Wnt, Notch, and Hedgehog

Extensive studies have recently demonstrated that proteins related to morphogenic differentiation processes may also exert substantial regulatory effects on the pro-fibrotic SSc pathways [143,144,145]. In the following sections, we will briefly review the pro-fibrotic effects induced by Wnt, Hedgehog, and Notch proteins. The extensive morphogenetic effects of these proteins will not be discussed because these effects are beyond the scope of the current review.

### 4.1. Wnt Signaling

The Wnt proteins comprise a large family of secreted glycoproteins with complex canonical and non-canonical intracellular signaling pathways that play crucial roles during embryonic development and organogenesis. Wnt proteins and pathways have been recently implicated in the pathogenesis of numerous diseases, including SSc and other fibrotic diseases [146,147,148]. Indeed, it has been shown that the secreted Frizzled Receptor Protein 4 (SFRP4), a Wnt-ligand-binding molecule, is increased in SSc, and its serum levels correlate with the extent and severity of SSc skin and lung fibrosis [148]. TGF-β appears to be the major factor activating the canonical Wnt pathway in fibrotic diseases, an effect probably mediated by a decrease in the potent Wnt pathway inhibitor. Based on these observations, it has been recently suggested that the inhibition of the Wnt pathways is currently being investigated as a potential therapeutic target in SSc and other fibrotic diseases [149,150,151].

### 4.2. Hedgehog and Notch Signaling

The Hedgehog (Hh) and Notch proteins are members of a large group of proteins collectively known as morphogens owing to their crucial roles in cell fate decisions during morphogenesis and embryonic development [152]. The involvement of these proteins in a broad spectrum of disorders, ranging from neurological diseases to inflammatory and immunological diseases and cancer, is just becoming apparent [153,154,155]. Of relevance to the pathogenesis of SSc are the observations that pro-fibrotic cytokines, including TGF-β, PDGF, and Wnt, drive Hh overexpression and that Hh-stimulated resting fibroblasts differentiate into myofibroblasts, causing an accumulation of collagen and dermal fibrosis [156,157,158]. Indeed, a recent study demonstrated that the antifibrotic effects of pirfenidone in SSc-ILD are mediated by inhibition of the Hh signaling pathway [159].

Regarding Notch, recent studies have shown that infiltrating T cells expressing the Jag-1 ligand in SSc skin might activate Notch signaling in dermal fibroblasts, leading to their transition to myofibroblasts and resulting in increased expression of ECM genes and production of their corresponding macromolecules [160]. Although Notch signaling may be induced under hypoxic conditions or by TGF-β, the detailed molecular mechanisms responsible for its pro-fibrotic effects and its potential role in the SSc-associated fibrotic process have not been fully elucidated [161,162,163,164].

In summary, TGF-β and growth factors play a major role in fibrosis in SSc patients. Table 1 summarizes the role of the growth factors mentioned in this manuscript. Many of these growth factors have context-dependent mechanisms of action, and their roles may be very different in different tissues. This may be at least partially responsible for the controversial clinical outcomes of studies inhibiting those factors. However, expanding the knowledge of the regulation and mechanism of action will help to find more effective treatment options for patients with SSc.

## Figures and Tables

**Table 1:** Growth factors and regulatory pathways in systemic sclerosis.

Growth Factor(s)	Receptor(s)	Target Cell(s)	Effect in SSc	References
TGF-β (TGF-β1,2,3)	TβRI (ALK5 in fibroblasts, ALK1 in endothelial cells), TβRII, TβRIII, integrins (αvβ6)	Fibroblasts, endothelial cells, epithelial cells, adipocytes, vascular smooth muscle cells	Strongly pro-fibrotic: ↑ collagen I/III, TIMPs, ECM proteins; ↓ MMPs; induces fibroblast→myofibroblast transdifferentiation; EndoMT, EMT; angiogenesis (context-dependent)	[29,30,31,32,33,34,35,36,37,38,39,40,41,42,43,44,45,46,47,48,49,50,51,52,53,54,55,56,57,58,59,60,107,108,109,125,126,127,133,134,135,136,137,138,139,140]
CTGF (CCN2)	Interacts with TGF-βRII, integrins, EGFR, ECM proteins	Fibroblasts, endothelial cells, vascular smooth muscle cells	Mediates downstream TGF-β fibrogenic effects; fibroblast proliferation, myofibroblast differentiation, vascular changes	[67,68,69,70,71,72,73,74,75,76,77]
PDGF (A–D)	PDGFR-α, PDGFR-β (RTKs)	Fibroblasts, vascular smooth muscle cells	Potent mitogen; ↑ fibroblast and SMC proliferation; promotes pulmonary fibrosis and PAH; PDGFR-α autoantibodies activate fibroblasts → ROS, ERK pathway	[78,79,80,81,82,83]
FGFs (esp. FGF-2)	FGFRs (RTKs)	Fibroblasts, endothelial cells	Mitogenic, angiogenic; ↑ FGF-2 in SSc skin; context-dependent: pro-fibrotic or antifibrotic (FGF-1 inhibits TGF-β1 effects)	[84,85,86,87,88]
VEGF (VEGF-A, VEGF165, VEGF165b)	VEGFR-1, VEGFR-2	Endothelial cells, fibroblasts	↑ in SSc serum; correlates with fibrosis and capillary loss; VEGF165b isoform antiangiogenic → defective angiogenesis	[89,90,91,92,93,94,95,96,97,98]
IGFs (IGF1,IGF-2)	IGF-1R, regulated by IGFBPs	Fibroblasts, endothelial cells, skin & lung cells	↑ IGF-1 and IGFBP-3 in SSc serum; IGF-2 promotes fibroblast activation via PI3K/JNK; ↑ collagen and FN	[99,100,101,102,103,104]
PKC-δ	Downstream of TGF-βR; intracellular kinase	Fibroblasts, endothelial cells, SMCs, mesangial cells	Modulates TGF-β/Smad signaling; ↑ collagen expression via phosphorylation cascades; higher in SSc fibroblasts	[105,106,107,108,109]
PI3K Pathway	PI3K receptors; interacts with endothelin receptor (ETA)	Fibroblasts, immune cells	↑ PI3K activity in SSc platelets; regulates COL1A2; Rac/PI3K pathway promotes myofibroblast activation	[106,107,108,109]
Lysophosphatidic Acid (LPA)	LPA receptors (LPARs, GPCR family)	Fibroblasts, endothelial cells	Potent profibrotic mitogen; activates Rho/ROCK pathway → myofibroblast differentiation; amplification loop with IL-6	[110,111,112,113,114,115,116,117,118,119]
Caveolin-1	Regulates TGF-βR trafficking	Fibroblasts, endothelial cells	Caveolin-1 loss → uncontrolled TGF-β activation; ↓ Caveolin-1 induces EndoMT; restoration prevents fibrosis and PAH in models	[120,121,122,123,124,125,126,127,128,129,130]
JAK/STAT Pathway	JAK kinases, STAT proteins	Fibroblasts, immune cells	Amplifies TGF-β and cytokine (IL-4, IL-6, IL-13) effects; promotes fibroblast→myofibroblast transition	[131,132,133,134]
PPAR-γ	Nuclear receptor (PPAR family)	Fibroblasts, adipocytes	Normally antifibrotic; ↓ PPAR-γ in SSc → exaggerated fibrosis; agonists restore balance	[135,136,137,138,139,140,141,142]
Wnt Pathway	Frizzled receptors, LRP5/6 (canonical)	Fibroblasts, endothelial cells	TGF-β activates canonical Wnt → profibrotic; ↑ SFRP4 in SSc serum correlates with fibrosis severity	[147,148,149,150,151,152]
Hedgehog (Hh)	Patched (PTCH), Smoothened (SMO)	Fibroblasts	Overexpression induced by TGF-β, PDGF, Wnt; promotes fibroblast→myofibroblast differentiation, ↑ collagen	[157,158,159,160]
Notch	Notch receptors (Notch1–4), ligands (Jag-1, DLL)	Fibroblasts, T cells	Jag-1+ T cells activate Notch in dermal fibroblasts → myofibroblast transition; ↑ ECM production	[161,162,163,164]

## Data Availability

No new data were created or analyzed in this study. Data sharing is not applicable to this article.
